# A modeling approach for estimating hydrogen sulfide solubility in fifteen different imidazole-based ionic liquids

**DOI:** 10.1038/s41598-022-08304-y

**Published:** 2022-03-15

**Authors:** Jafar Abdi, Masoud Hadipoor, Seyyed Hamid Esmaeili-Faraj, Behzad Vaferi

**Affiliations:** 1 Faculty of Chemical and Materials Engineering, Shahrood University of Technology, Shahrood, Iran; 2 Department of Petroleum Engineering, Ahwaz Faculty of Petroleum Engineering, Petroleum University of Technology (PUT), Ahwaz, Iran; 3 Department of Chemical Engineering, Shiraz Branch, Islamic Azad University, Shiraz, Iran

**Keywords:** Chemical engineering, Environmental sciences

## Abstract

Absorption has always been an attractive process for removing hydrogen sulfide (H_2_S). Posing unique properties and promising removal capacity, ionic liquids (ILs) are potential media for H_2_S capture. Engineering design of such absorption process needs accurate measurements or reliable estimation of the H_2_S solubility in ILs. Since experimental measurements are time-consuming and expensive, this study utilizes machine learning methods to monitor H_2_S solubility in fifteen various ILs accurately. Six robust machine learning methods, including adaptive neuro-fuzzy inference system, least-squares support vector machine (LS-SVM), radial basis function, cascade, multilayer perceptron, and generalized regression neural networks, are implemented/compared. A vast experimental databank comprising 792 datasets was utilized. Temperature, pressure, acentric factor, critical pressure, and critical temperature of investigated ILs are the affecting parameters of our models. Sensitivity and statistical error analysis were utilized to assess the performance and accuracy of the proposed models. The calculated solubility data and the derived models were validated using seven statistical criteria. The obtained results showed that the LS-SVM accurately predicts H_2_S solubility in ILs and possesses R^2^, RMSE, MSE, RRSE, RAE, MAE, and AARD of 0.99798, 0.01079, 0.00012, 6.35%, 4.35%, 0.0060, and 4.03, respectively. It was found that the H_2_S solubility adversely relates to the temperature and directly depends on the pressure. Furthermore, the combination of OMIM^+^ and Tf_2_N^-^, i.e., [OMIM][Tf_2_N] ionic liquid, is the best choice for H_2_S capture among the investigated absorbents. The H_2_S solubility in this ionic liquid can reach more than 0.8 in terms of mole fraction.

## Introduction

In the recent century, the need for fossil fuels has risen due to the high levels of energy required for the rapid industrialization of the world^1^. The extraction of oil and gas from underground fields and their combustion for generating heat/energy^2^ has undesired environmental effect^3^ and is accompanied by the production of large amounts of undesired pollutants^4–6^, mainly carbon monoxide (CO)^7^, carbon dioxide (CO_2_)^8,9^, sulfur dioxide (SO_2_)^10^, and hydrogen sulfide (H_2_S)^11,12^. The most widely used method for removing these gases is absorption^13^. The absorption processes can help humans meet environmental standards and attenuate the global warming issue^14^. Nowadays, the absorption process using alkanolamine- based solvent is one of the most developed and industrially interesting approaches^15^. However, the loss of mono-ethanolamine, diethanolamine, N-methyl-diethanolamine, and di-isopropanol amine creates environmental problems, and they have also produced some highly corrosive byproducts^16,17^. As an alternative and promising approach, scientists have investigated ionic liquids (ILs)^18–20^. Ionic liquids are comprised of cations and anions and have an asymmetric organic cation structure, which results in being liquid at room temperature^21^. Ionic liquids possess outstanding thermal stability and a superior ability to solve organic and non-organic problems^18,19^. These features are highly attributed to their cation and anion particles. Cations and anions of ionic liquids can be easily modified to make them suitable for many specific applications^19^. Furthermore, having an insignificant vapor pressure, ILs have been considered promising candidates for sweetening processes with the minimum environmental effect and solvent loss^22^. A central factor that must be appraised in gas sweetening processes is the solubility of gases in liquids under various dominated operational conditions^23,24^. Although many references in the literature have calculated or experimentally obtained the CO_2_ solubility in ILs^16–18,25–27^, authentic data representing the H_2_S solubility in ILs is scarce. Therefore, developing robust predictive models is crucial for precisely and expeditiously estimation of H_2_S solubility data in various operational conditions. In recent years, numerous investigations have been performed to evaluate gas solubility in different ILs. Shariati and Peters^28^ implemented the Peng–Robinson (PR) equation of state to obtain the solubility of CHF_3_ in [C_2_mim][PF_6_] under various pressures and temperatures. Kroon et al.^29^ estimated the solubility of CO_2_ in different ILs at high pressures less than 100 MPa. Wang et al.^30^ used the square-well chain fluid equation of state (EoS) to assess several gases’ solubility in ILs. Researchers have used numerous EoSs and methods to evaluate both H_2_S and CO_2_ solubility in ILs^25,31,32^. Nevertheless, none of the above-mentioned approaches could be generalized to different systems. As a result, a range of more general approaches must be applied to forecast gas solubility in ILs. Recently, many intelligent methods, such as artificial neural networks (ANNs)^33^ have been applied for predicting various properties in chemical engineering, including crstallinity^34,35^, thermal conductivity^36,37^, viscosity^38^, heat capacity^39^, and solubility of different gases in solutions^40,41^.

Predication of CO_2_ solubility in ILs is not an exception, and many soft computing methods have been used for this purpose^42,43^. In contrast, we found that limited investigations have been done regarding the estimation of H_2_S solubility in ILs using artificial intelligence (AI) models, and further research activities are needed. In the present work, we implement different intelligence models, including multilayer perceptron neural network (MLPNN), adaptive neuro-fuzzy inference system (ANFIS), least-squares support vector machine (LS-SVM), radial basis function neural network (RBFNN), cascade feedforward neural network (CFFNN), and generalized regression neural network (GRNN) for accurate estimation of the H_2_S solubility in ILs. For this aim, fifteen ILs under different pressure and temperature conditions are investigated. In addition, the preciseness and reliability of the best method have been compared with the UNIFAC EoS. Several graphical and statistical techniques are used to evaluate the performance of the developed models, and the relevancy factor investigates the effect of each input parameter. Moreover, the trend analysis is carried out to assess the capability of the proposed models in detecting the physical trend between the H_2_S solubility and different temperatures and pressures. At last, the Leverage approach is made to check the validity of the data and feasible region of the best-proposed model.

## Theoretical background

### Multilayer perceptron neural network

A feedforward MLPNN has three layers of input, interiors, and output^44–46^. MLPNN benefits from a unique training approach known as the backpropagation, and the utilized activation functions in this method are non-linear^47^. The three most common types of activation functions are specified as follows^37,48^:

1 $${\text{Linear:}}\,\,\,\,f\left( x \right) = x$$

2 $${\text{Logarithm sigmoid:}}\,\,\,\,f\left( x \right) = \frac{1}{{1 + e^{ - x} }}$$

3 $${\text{Tangent sigmoid:}}\,\,\,\,f\left( x \right) = \frac{{e^{x} - e^{ - x} }}{{e^{x} + e^{ - x} }}.$$

### Adaptive neuro-fuzzy inference system

A combination of ANN with fuzzy logic will result in the emergence of ANFIS systems. Typically, two common structures for FIS approaches exist: (a) Mamdani et al. and (b) Takagi–Sugeno^36,49^. What is specific about Mamdani et al. method^50^ is that a list of if–then rules must be defined for the fuzzy inference system, while the fussy interface proposed by Takagi–Sugeno creates its own rules based on the intrinsic features of the provided experimental data to the modeling endeavor. If the output data is nonlinearly dependent on the input data, Takagi–Sugeno ANFIS method will be more useful. Five distinct layers are a typical architecture for the ANFIS structure^51^. The Fuzzification layer is the first layer in which the conversion of inputted data into linguistic data occurs. The fuzzification process will be done utilizing the defined membership functions. The second layer is used for the model validation by computing a range of parameters known as the firing strengths. The estimated firing strengths are normalized in the next layer, and the fourth layer is responsible for representing outputs’ linguistic terms. Ultimately, all rules attributed to any individual output are combined in the fifth layer^50^.

### Least square-support vector machine

As a robust method for pattern recognition^52^ and regression^53^, the LS-SVM is a widely-used and well-developed method. The SVM formulates the function as is given in Eq. (4).

4 $$f \left( x \right) = w^{T} \left( x \right)\varphi \left( x \right) + b$$

where the output layer’s transposed vector is denoted by *w*^*T*^, the kernel function and bias are given as *φ(x*) and b, respectively^54,55^. The size of the input data set and the output ensemble are the determining factors for the SVM’s dimension. The parameters of *w* and *b* are then determined by the cost function, given in Eq. (5)^54^.

5 $$Cost\,function = \frac{1}{2}w^{T} + c\mathop \sum \limits_{k = 1}^{N} \left( {\xi_{k} - \xi_{k}^{*} } \right)$$

The reliable results are possible to achieve by minimizing the cost function considering the following constraints^54^:

6 $$\left\{ \begin{gathered} y_{k} - w^{T} \varphi \left( {x_{k} } \right) - b \le \varepsilon + {\upxi }_{{\text{k}}} , k = 1, 2, \ldots , N \hfill \\ w^{T} \varphi \left( {x_{k} } \right) + b - y_{k} \le \varepsilon + {\upxi }_{k}^{*} , k = 1, 2, \ldots , N \hfill \\ \xi_{k} , \xi_{k}^{*} \ge 0 \hfill \\ \end{gathered} \right.$$

where the k_th_ inputted data and its corresponding output are shown by *x*_*k*_* and y*_*k*_, respectively. In this formulation, *ε* stands for the accurateness of the function results, and the maximum acceptable errors are given by $\xi_{k}$ and $\xi_{k}^{*}$ Indicates the slack variable. The deviations from *ε* are determined by c values.

### Radial basis function neural network

RBF neural networks are robust predicting methods that use a simpler structure in comparison to MLP networks, the learning step in them is much faster than the MLP’s learning procedure^56^. Like major artificial neural networks, RBF has three layers: the input layer, the interior layers, and the result layer. The radial basis function is applied to the nodes of hidden layers. Using a linear optimization mechanism, the RBFNN will return precise results when the least mean square error is achieved. Despite all existing similarities between MLPNN and the RBFNN structures, RBFNN utilizes a complex RBF function for hidden layers^36^.

### Cascade fee-forward neural network

The implemented CFFNN in this study could be contemplated as a type of feedforward neural network where the input neurons are connected to all neurons located in the following layers^57,58^. A range of various learning algorithms is applied to CFFNN models. As one of the most general formulations, the gradient descent algorithm with the momentum is introduced as follows^59^:

7 $$\Delta w\left( {i + 1} \right) = - \alpha \frac{{\partial E_{P} }}{\partial w} + \mu \Delta w\left( i \right) + \gamma e_{s} , \Delta b\left( {i + 1} \right) = - \alpha \frac{{\partial E_{P} }}{\partial b} + \mu \Delta b\left( i \right) + \gamma e_{s}$$

where the weight of neurons is denoted by $w$, the learning pace is shown by α, and bias and the number of training steps are given by b and i, respectively. In these formulations, the momentum parameter is presented by $\mu$, and the deviation of outputs from the modeling target is represented by $\gamma$^60^. Although the updating algorithm for weight factors (given in Eq. 4) is precise, it is just applicable to a small ensemble of data. The weights updating formulation with two terms (i.e., the formulation without $\gamma e_{s}$) is a better choice for modeling of large-scale databanks. Equation (8) shows that the cost function is defined by summation of the square error.

8 $$SSE = \mathop \sum \limits_{p = 1}^{n} E_{p} = \mathop \sum \limits_{p = 1}^{n} \left( {t_{p} - o_{p} } \right)^{2}$$

where the target and the output patterns are shown by t_p_ and o_p_. The training procedure will not stop unless a pre-defined desirable sum of square errors is obtained^61^.

### Generalized regression neural network

In utilizing the GRNN predictive method, there is no need for an iterative training process^13^. Instead, between the output and input vectors, any possible arbitrary functions are approximated. In addition to that, this approach is consistent because as larger datasets are fed to the model, the model return more precise results^62^. Such as the problems solved by the standard regression methods, the GRNN model is also suitable for predicting variables that are intrinsically continuous^62^. According to the definition of this method, the best and most accurate result for a dependent variable (y) will be obtained when an independent variable x and the training dataset are given, and the model commences minimizing the mean-squared error for the given x data points^62^.

## Experimental data acquisition and preliminary analysis

### Data gathering

In the current study, a collection of 792 datasets regarding to H_2_S solubility in fifteen different ionic liquids, including [OMIM][Tf_2_N], [OMIM][PF_6_], [HMIM][PF_6_], [BMIM][Tf_2_N], [HMIM][Tf_2_N], [EMIM][Tf_2_N], [HOeMIM][Tf_2_N], [BMIM][BF_4_], [BMIM][PF_6_], [EMIM][PF_6_], [EMIM][eFAP], [HOeMIM][OTF], [HOeMIM][PF_6_], [HEMIM][BF_4_], [EMIM][EtSO_4_] were assembled (the full form of these ionic liquids are introduced in Table 1). The range of operating conditions, i.e., pressure (P) and temperature (T), ionic liquid inherent characteristics, i.e., critical pressure (P_c_), critical temperature (T_c_), and the acentric factor (ω) are listed in Tables 1 and 2. The range of absorbed hydrogen sulfide by different ionic liquids as the dependent variable is also reported in Table 1. Indeed, these variables are enough to derive a global model for determining the amount of captured H_2_S in ILs. The gathered data points were divided into two main subsets, including training (85% of the datasets) and testing (15% of the datasets). These groups have been used in a systematic trial-and-error procedure to find the optimal configuration of the model structures and evaluate their performances.

**Table 1:** The range of operating conditions during absorbing H_2_S molecules by different ionic liquids.

Ionic liquid (Full name)	Abbreviation	Temperature (K)	Pressure (bar)	Solubility (Mole fraction)	Number of data	References
1-Ethyl-3-methylimidazolium ethylsulfate	[EMIM][EtSO_4_]	303.15–353.15	1.14–12.70	0.012–0.118	36	^17^
1-Ethyl-3-methylimidazolium hexafluorophosphate	[EMIM][PF_6_]	333.15–363.15	1.45–19.33	0.032–0.359	40	^25^
1-Ethyl-3-methylimidazolium bis(trifluoromethylsulfonyl)imide	[EMIM][Tf_2_N]	303.15–353.15	1.08–16.86	0.049–0.609	42	^25^
1-Ethyl-3-methylimidazolium tris(pentafluoroethyl) trifluorophosphate	[EMIM][eFAP]	303.15–353.15	0.58–19.42	0.022–0.592	79	^26^
1-Hexyl-3-methylimidazolium bis(trifluoromethanesulfonyl)imide	[HMIM][Tf_2_N]	303.15–353.15	0.69–20.17	0.029–0.701	87	^16,18^
1-Hexyl-3-methylilmidazolium hexafluorophosphate	[HMIM][PF_6_]	303.15–343.15	1.11–11.00	0.050–0.499	67	^16^
1-Butyl-3-methylimidazolium tetrafluoroborate	[BMIM][BF_4_]	303.15–343.15	0.61–8.36	0.030–0.354	42	^63^
1-Butyl-3-methylimidazolium hexafluorophosphate	[BMIM][PF_6_]	298.15–403.15	0.69–96.30	0.016–0.875	81	^63,64^
1-Butyl-3-methylimidazolium bis(trifluoromethylsulfonyl)imide	[BMIM][Tf_2_N]	303.15–343.15	0.94–9.16	0.051–0.510	44	^63^
1-Octyl-3-methylimidazolium bis(trifluoromethylsulfonyl)imide	[OMIM][Tf_2_N]	303.15–353.15	0.94–19.12	0.063–0.735	47	^18^
1-n-Octyl-3-methylimidazolium hexafluorophosphate	[OMIM][PF_6_]	303.15–353.15	0.85–19.58	0.046–0.697	48	^31^
1-(2-Hydroxyethyl)-3-methylimidazolium bis(trifluoromethylsulfonyl)imide	[HOeMIM][Tf_2_N]	303.15–353.15	1.56–18.32	0.057–0.572	41	^27^
1-(2-Hydroxyethyl)-3-methylimidazolium trifluoromethanesulfonate	[HOeMIM][OTf]	303.15–353.15	1.06–18.39	0.035–0.548	41	^27^
1-(2-Hydroxyethyl)-3-methylimidazolium hexafluorophosphate	[HOeMIM][PF_6_]	303.15–353.15	1.34–16.85	0.034–0.462	47	^27^
1-(2-Hydroxyethyl)-3-methylimidazolium tetrafluoroborate	[HEMIM][BF_4_]	303.15–353.15	1.21–10.66	0.020–0.247	50	^23^

**Table 2:** The critical temperature, pressure, and acentric factors of ILs used in this study.

Abbreviation	T_c_ (K)	P_c_ (bar)	ω	References
[EMIM][EtSO_4_]	1061.1	40.4	0.3368	^65^
[EMIM][PF_6_]	663.5	19.5	0.6708	^65^
[EMIM][Tf_2_N]	1244.9	32.6	0.1818	^65^
[EMIM][eFAP]	830. 7	100.3	1.5099	^66^
[HMIM][Tf_2_N]	876.2	22.2	1.3270	^65^
[HMIM][PF_6_]	754.3	15.5	0.8352	^65^
[BMIM][BF_4_]	632.3	20.4	0.8489	^65^
[BMIM][PF_6_]	708.9	17.3	0.7553	^65^
[BMIM][Tf_2_N]	1265	27.6	0.2656	^65^
[OMIM][PF_6_]	800.1	14.0	0.9069	^19^
[OMIM][Tf_2_N]	923.0	18.7	1.3310	^65^
[HOeMIM][Tf_2_N]	1297	33.1	0.5171	^27^
[HOeMIM][OTf]	1059.1	36.7	0.6526	^27^
[HOeMIM][PF_6_]	766.9	20.2	1.0367	^27^
[HEMIM][BF_4_]	691.9	24.7	1.1643	^67^

### Outlier detection

Outliers are typically an inevitable part of every dataset; therefore, eliminating outliers is extremely important for good quality and reliable modeling. Outliers can drastically plummet the model’s accuracy and robustness. The current study reaps the outstanding rewards of utilizing a combination of Leverage and the Hat matrix methods according to the below equation^68^:

9 $$H = X\left( {X^{T} X} \right)^{ - 1} X^{T}$$

where X is the matrix of independent variables in the [n × m] shape, i.e., numbers of features × numbers of measurements. The process of outlier detection is done using a William plot. Calculation, normalization, and illustration of residual values with respect to the hat value are performed by developing this plot. Simultaneously, a warning leverage value (H*) is calculated using the following expression^69^:

10 $$H^{*} = \frac{{3\left( {n + 1} \right)}}{m}.$$

### Statistical criteria for model assessment

Once the models are developed, the accuracy can be evaluated by various statistical approaches to determine their robustness. In the current investigation, the following criteria were utilized for assessing models’ accuracy^70,71^:

11 $${\text{Root mean square error }}\left( {{\text{RMSE}}} \right) = \left( {\frac{{\mathop \sum \nolimits_{i = 1}^{N} (y_{i, pred.} - y_{i,exp.} )^{2} }}{N}} \right)^{1/2}$$

12 $${\text{Coefficient of determination }}\left( {{\text{R}}^{2} } \right) = 1 - \frac{{\mathop \sum \nolimits_{i = 1}^{n} (y_{i, pred.} - y_{i,exp.} )^{2} }}{{\mathop \sum \nolimits_{i = 1}^{n} (y_{i, pred.} - \overline{y}_{i,exp.} )^{2} }}$$

13 $${\text{Absolute average relative deviation }}\left( {{\text{AARD\% }}} \right) = \frac{100}{N}\mathop \sum \limits_{i = 1}^{N} \frac{{\left| {y_{i, pred.} - y_{i,exp.} } \right|}}{{y_{i,exp.} }}$$

14 $${\text{Mean absolute error }}\left( {{\text{MAE}}} \right) = \frac{{\mathop \sum \nolimits_{i = 1}^{N} \left| {y_{i, pred.} - y_{i,exp.} } \right|}}{N}$$

15 $${\text{Relative absolute error }}\left( {{\text{RAE\% }}} \right) = \frac{{100 \times \mathop \sum \nolimits_{i = 1}^{N} \left| {y_{i, pred.} - y_{i,exp.} } \right|}}{{\mathop \sum \nolimits_{i = 1}^{N} \left| {\overline{y} - y_{i,exp} } \right|}}$$

16 $${\text{Root relative squared error }}\left( {{\text{RRSE\% }}} \right) = 100 \times \sqrt {\frac{{\mathop \sum \nolimits_{i = 1}^{N} \left( {y_{i, pred.} - y_{i,exp.} } \right)^{2} }}{{\mathop \sum \nolimits_{i = 1}^{N} \left( {\overline{y} - y_{i,exp.} } \right)^{2} }}}$$

17 $${\text{Mean square errors }}\left( {{\text{MSE}}} \right) = \frac{1}{N}\mathop \sum \limits_{i = 1}^{N} \left( {y_{i,pred.} - y_{i, exp.} } \right)^{2}$$

In these equations, employed for statistical evaluation of the results, $y_{i,exp.}$ and $y_{i, pred.}$ show the experimentally measured and the predicted H_2_S solubilities, respectively. The notation $\overline{y}$ is the average value of $y_{i,exp.}$, and N stands for the number of data points.

## Result and discussion

### Development phase

All considered machine learning mthods^72–74^ have some parameters that need to be tuned using historical data of a given problem and an optimization algorithm^75^. This research utilizes 792 experimental data of H_2_S solubility in fifteen ILs versus pressure, temperature, acentric factor, critical pressure, and temperature. The collected databank was randomly split into 673 training and 119 testing datasets.

In the training stage, a machine learning method receives the numerical values of independent as well as dependent variables, while its parameters are unknown^76–78^. The intelligent model estimates the H_2_S solubilities from the available independent variables. The deviation between these estimated values and actual H_2_S solubilities are then needed to be minimized by an optimization algorithm. Indeed, the optimization algorithm continuously updates the parameters of a machine learning method to converge to this minimum value.

In the testing stage, a trained machine learning method receives the independent variables only and calculates the H_2_S solubility helping the adjusted parameters. The independent variables and machine learning parameters are known in the testing stage, while the dependent variables are unknown.

**Figure 1:**
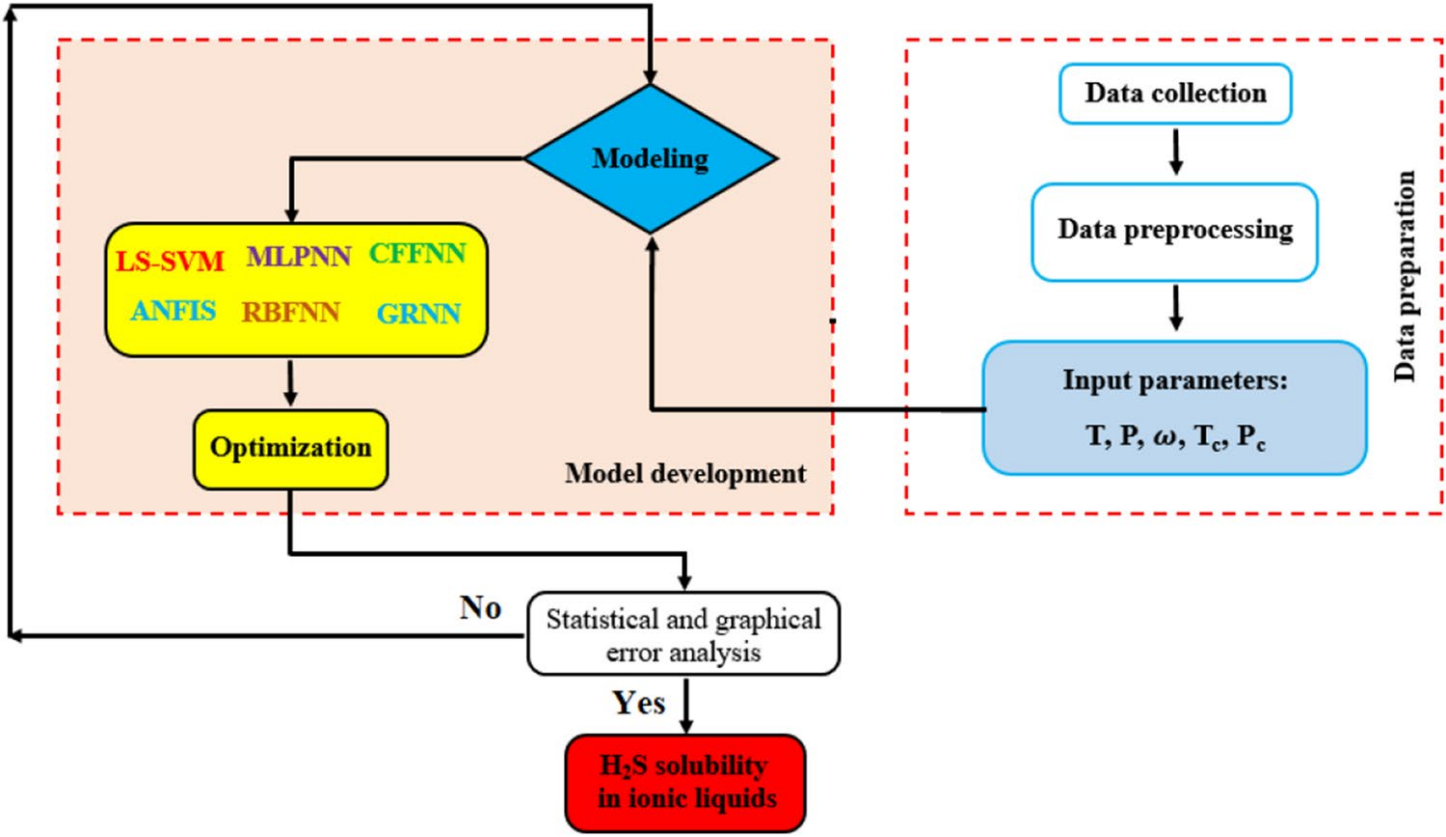
General sketch for development of the proposed models.

The accuracy of all machine learning methods in the training and testing stages has been monitored using different statistical indices [i.e., Eqs. (11–17)]. Then, it is possible to find the best model using the ranking analysis. Figure 1 represents a general flowchart for model development in the present study.

Table 3 shows the complete information about the applied trial-and-error procedure during model development. This table shows the numbers of hidden neurons for the MLPNN, CFFNN, and RBFNN, spread factor for the RBFNN and GRNN, cluster radius for the ANFIS, and kernel type for the LS-SVM model is the deciding features in the trial-and-error analyses. Table 3 also presents the cumulative numbers of the developed model for each machine learning class. Generally, 740 models are developed in this study.

**Table 3:** General information about the model development phase.

Model	Decision features	Range of decision features	Numbers of iteration	Numbers of developed models
ANFIS	Cluster radius training algorithm	0.5–1 (10 values) Backpropagation and hybrid	10 per cluster radius	200
LS-SVM	Kernel types	Linear, polynomial, Gaussian	30 per kernel function	90
MLPNN	Numbers of hidden neurons	1–9 (9 values)	10 per hidden neuron	90
CFFNN	Numbers of hidden neurons	1–8 (8 values)	10 per hidden neuron	80
RBFNN	Numbers of hidden neurons Spread values	1–9 (9 values) 10^–6^-10 (20 values)	20 per hidden neuron	180
GRNN	Spread values	10^–6^-10 (100 values)	One per spread factor	100

### Assessment phase

#### Statistical analyses

After the model development phase, monitoring their accuracy in the training and testing stages employing various statistical criteria is necessary. In this way, it is possible to find the most accurate model in each class using the ranking analysis. The prediction uncertainty of the most precise model in each category in terms of seven statistical criteria is summarized in Table 4. This table states that the cluster radius of 0.5 and Gaussian kernel are the best features for the ANFIS and LS-SVM paradigms. Furthermore, nine, six, and nine hidden neurons are the best topologies of the MLPNN, CFNN, and RBFNN models. The best spread factor for the RBFNN and GRNN models are 3.1579 and 0.00210, respectively. As this table shows, almost all intelligent models are sufficiently robust for estimating hydrogen sulfide solubility in various ionic liquid media. All models show the R^2^ values greater than 0.99, apart from RBFNN.

**Table 4:** Statistical evaluation of the best selected model in each class.

Model	Key feature	Stage	AARD%	MAE	RAE%	RRSE%	MSE	RMSE	R^2^
ANFIS	Cluster radius = 0.5	Training	5.20	0.0075	5.34	6.84	0.00014	0.01178	0.997657
		Testing	4.99	0.0075	5.93	7.62	0.00014	0.01180	0.997105
		Total	5.17	0.0075	5.42	6.94	0.00014	0.01178	0.997587
LS-SVM	Gaussian kernel	Training	3.89	0.0058	4.17	6.21	0.00011	0.01063	0.998073
		Testing	4.78	0.0070	5.45	7.26	0.00014	0.01165	0.997427
		Total	4.03	0.0060	4.35	6.35	0.00012	0.01079	0.997980
MLPNN	9 hidden neurons	Training	4.76	0.0067	4.85	6.60	0.00013	0.01131	0.997819
		Testing	3.55	0.0067	5.01	7.38	0.00014	0.01189	0.997363
		Total	4.58	0.0067	4.87	6.72	0.00013	0.01140	0.997746
CFFNN	6 hidden neurons	Training	4.95	0.0074	5.47	7.20	0.00014	0.01202	0.997408
		Testing	5.71	0.0069	4.33	7.26	0.00018	0.01340	0.997475
		Total	5.06	0.0073	5.27	7.21	0.00015	0.01224	0.997400
GRNN	Spread = 0.00210	Training	0.59	0.0011	0.81	2.66	0.00002	0.00460	0.999646
		Testing	25.72	0.0478	40.10	39.23	0.00344	0.05864	0.926456
		Total	4.36	0.0082	5.89	13.62	0.00053	0.02312	0.990788
RBFNN	9 hidden neurons, spread = 3.1579	Training	33.62	0.0469	33.62	35.99	0.00380	0.06161	0.932988
		Testing	26.51	0.0405	30.99	33.19	0.00285	0.05340	0.943624
		Total	32.55	0.0460	33.22	35.61	0.00365	0.06045	0.934448

#### Graphical inspection

**Figure 2:**
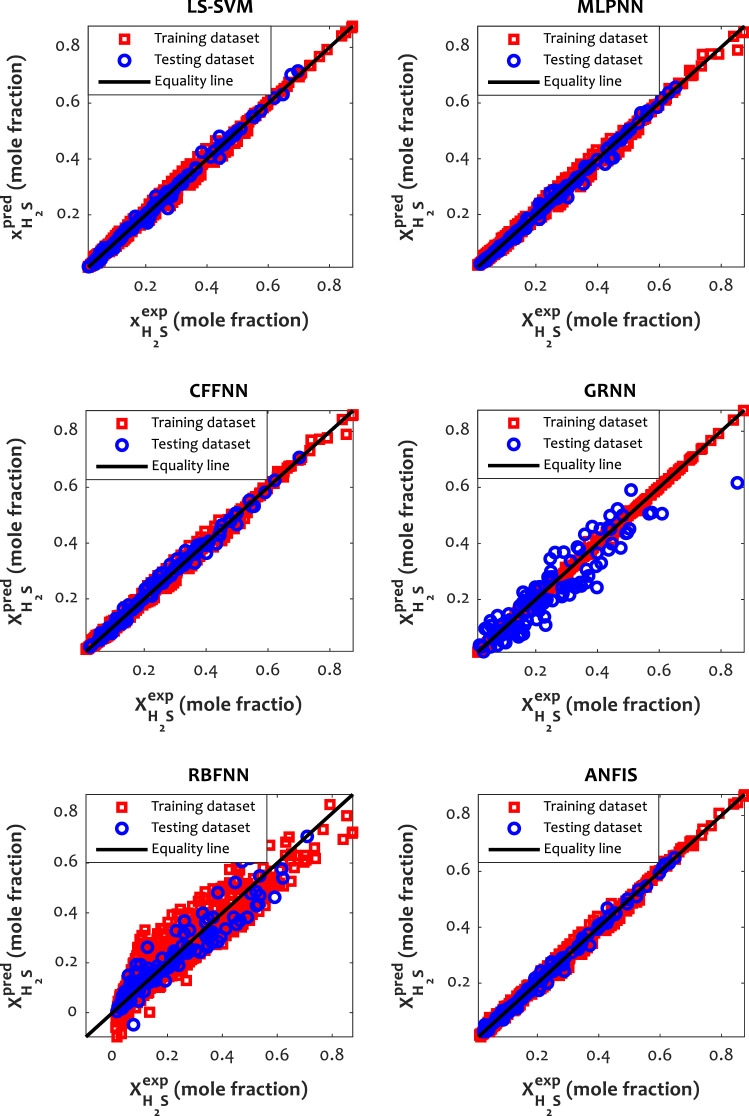
Cross plots of the best model in each class.

**Figure 3:**
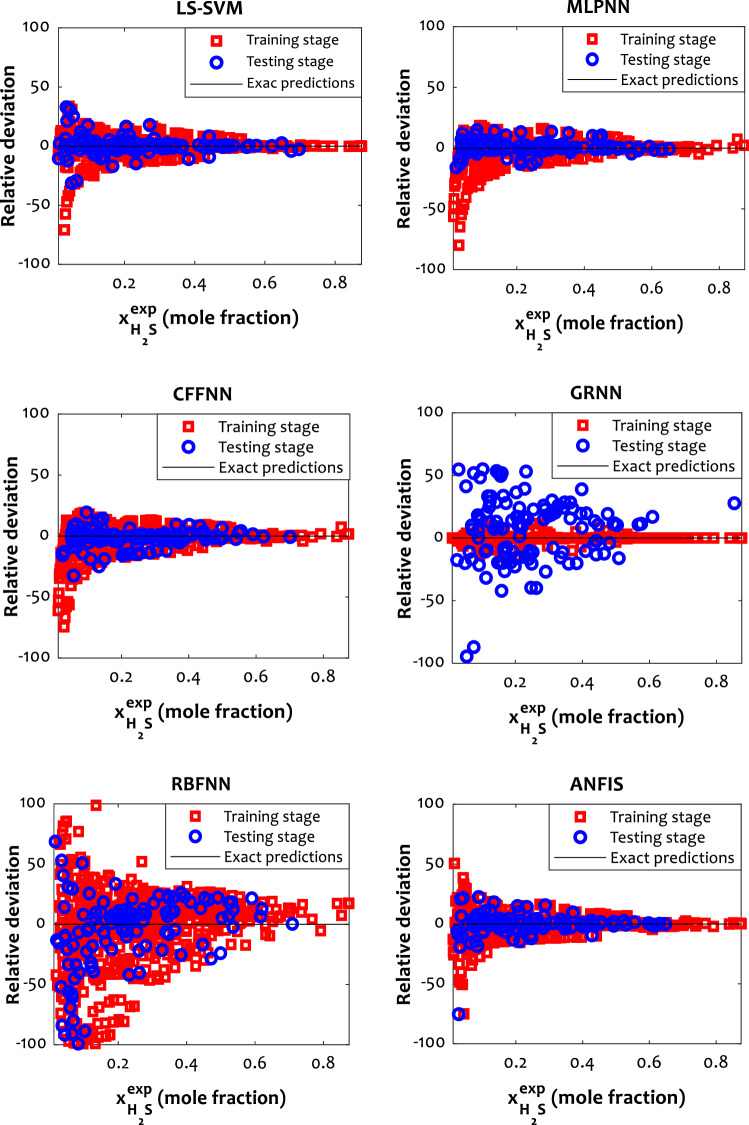
The relative deviations of the selected models for estimating the H_2_S solubility.

Different graphical inspections, such as cross-plot and distribution of residual errors, were performed to illustrate the efficiency of the developed models and compare their performances. Figure 2 shows the cross plots of all the implemented approaches and confirms an excellent agreement between experimental and predicted mole fractions of H_2_S in ILs due to the concentrated accumulation of the training and testing data around the unit slope line. In addition, the relative deviation of the investigated models from experimental data is depicted in Fig. 3. The error distribution provides a suitable visual comparison between the models’ performances. In this figure, LS-SVM, MLPNN, CFFNN, and ANFIS have small scattering to anticipate H_2_S solubility in various ILs, while the relative deviation of the training and testing data points for GRNN and RBFNN models exceed 40%. These findings confirm the obtained results in Table 4.

#### Ranking analysis

**Figure 4:**
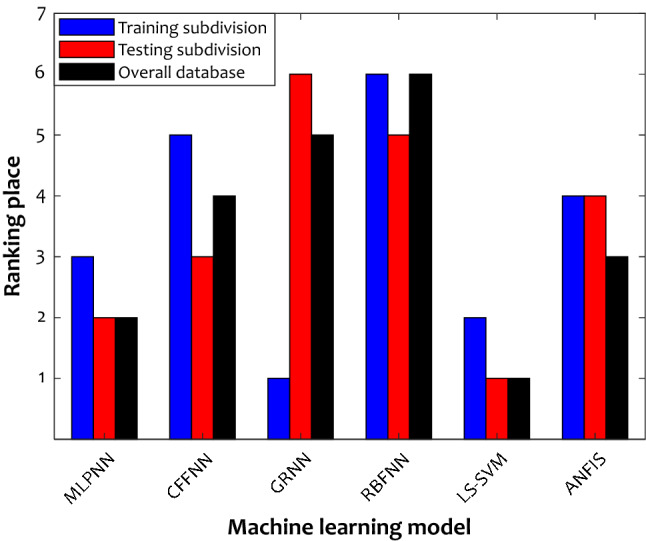
Developed models ranking based on seven different statistical indices.

The six models were selected before, and their accuracy in the training and testing stages and over the whole of the database was monitored using seven statistical matrices. It is hard to most accurate one through visual inspection. Therefore, the ranking analysis is employed to do so^40^. Figure 4 provides the results of model ranking in each stage based on the average values of the seven statistical criteria reported in Table 4. The GRNN model in the learning step is the best model; nevertheless, it shows the worst performance in the testing stage. This sharp contrast between the learning ability and the testing results indicates the overfitting of the GRNN model. On the other hand, the LS-SVM with the second-ranking in the training stage and the first ranks for the testing stage and over the whole database is the best model for predicting H_2_S solubility in ionic liquid media.

According to the results of Fig. 4, the developed predictive models can be summarily ranked in terms of their accuracy as follows: LS-SVM > MLPNN > ANFIS > CFFNN > GRNN > RBFNN.

**Figure 5:**
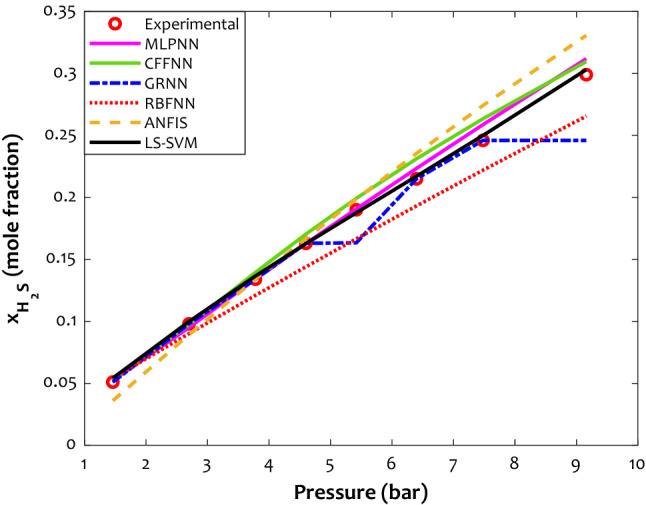
The prediction ability of the developed models for H_2_S solubility in [BMIM][Tf_2_N] (T = 343.15 K).

Figure 5 depicts the performance of different models to predict the experimental data of H_2_S solubility in [BMIM][Tf_2_N] ionic liquid versus pressure at 343.15 K. The LS-SVM is the most precise model for estimating the H_2_S solubility in ionic liquid media.

**Figure 6:**
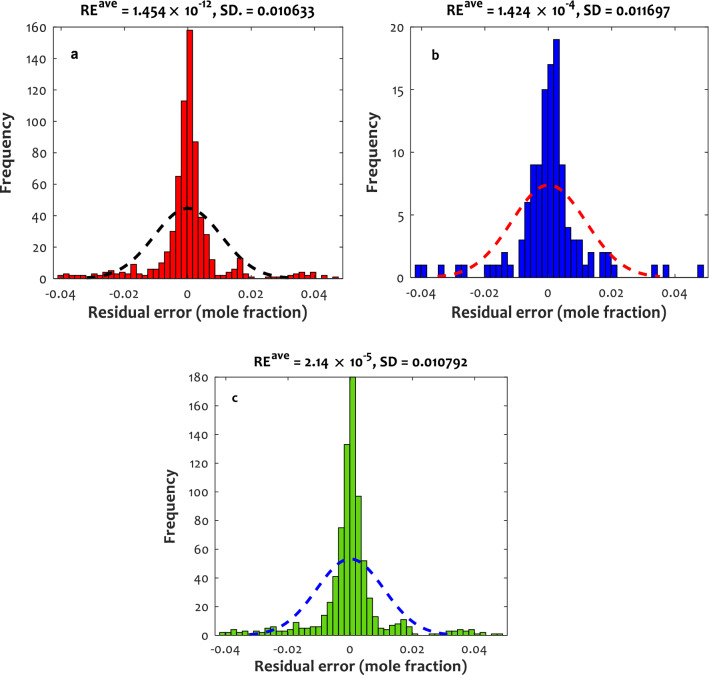
Histograms of the residual errors (RE) of the LS-SVM for predicting H_2_S solubility in different IL media (**a**) training group, (**b**) testing group, and (**c**) all datasets.

As mentioned earlier, the LS-SVM approach was selected as the best model. In order to better describe the excellent performance of the LS-SVM model, its residual errors (RE) versus the frequency are plotted in Fig. 6. Histograms related to the training, testing, and all data points reveal that the maximum frequency could be seen around residual errors of zero, and virtually all data points are predicted with − 0.05 < RE <  + 0.05.

#### Equations of state

**Figure 7:**
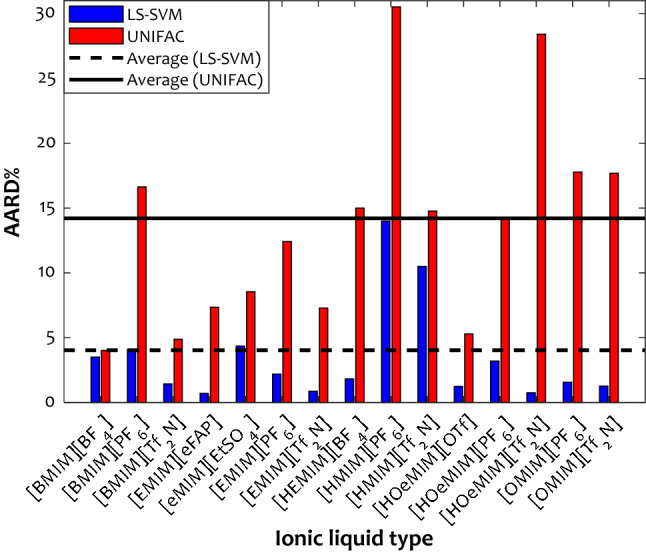
Comparison between LS-SVM and UNIFAC uncertainties^79^ to estimate the H_2_S solubility in various ILs.

The prediction uncertainty (in terms of AARD) of the LS-SVM and UNIFAC EoS^79^ to estimate the 792 collected H_2_S solubility in various IL media are compared in Fig. 7. It can be seen that the LS-SVM (AARD ~ 14%) and UNIFAC (AARD ~ 30%) show the maximum uncertainty for the H_2_S solubility in the [HMIM][PF_6_] ionic liquid. Although the UNIFAC has its second-highest AARD of 27% for [HOeMIM][Tf_2_N], the obtained value by the LS-SVM is about thirteen times lower (AARD ~ 2%). Indeed, the results predicted by the LS-SVM method are remarkably better than the UNIFAC results. The overall AARD% of UNIFAC for H_2_S solubility in all IL media is ~ 14%, while it is about 4% for the LS-SVM model. Excluding hydrogen sulfide solubility in the [HMIM][PF_6_] and [HMIM][TF_2_N] ionic liquids, the LS-SVM predicts all other systems with the AARD of lower than 5%. It confirms the LS-SVM excellent capability for a wide range of IL/H_2_S systems.

Table 5 presents the accuracy of the proposed LS-SVM model and other approaches reported in the literature based on AARD%. As can be observed, the implemented model in this work shows precise performance for estimating H_2_S solubility in ILs with AARD% less than that for other EoS models and intelligent algorithms. In addition, other reports have developed for a smaller number of ILs and data points compared to the present study.

**Table 5:** Comparison between the developed LS-SVM model and the available approaches in the literature for estimating H_2_S solubility in ILs based on AARD%.

Model	No. of data points	No. of ILs	AARD (%)	Ref
Peng-Robinson (kij = 0)	465	11	38.95	^80,81^
Soave–Redlich–Kwong (kij = 0)	465	11	36.43	^80,81^
Peng-Robinson	465	11	4.90	^80,81^
Soave–Redlich–Kwong	465	11	4.87	^80,81^
Peng-Robinson (kij = 0)	664	14	196.76	^66^
Peng-Robinson	664	14	8.35	^66^
RETM-CPA	317	5	4.41	^82^
SAFT-VR	225	6	5.44	^83^
Peng-Robinson-Two State	636	12	3.40	^84^
Peng-Robinson (kij = 0)	664	14	25.13	^85^
Peng-Robinson	664	14	3.67	^85^
FC-ELM	722	16	2.33	^86^
Gene expression programming	465	11	4.38	^80,81^
MLPNN	496	12	1.94	^87^
MLPNN	664	14	2.07	^66^
MLPNN	664	13	9.08	^88^
RBFNN	664	13	26.15	^88^
ANFIS	664	13	38.44	^88^
LS-SVM	792	15	4.02	This work

### Relevancy analysis

**Figure 8:**
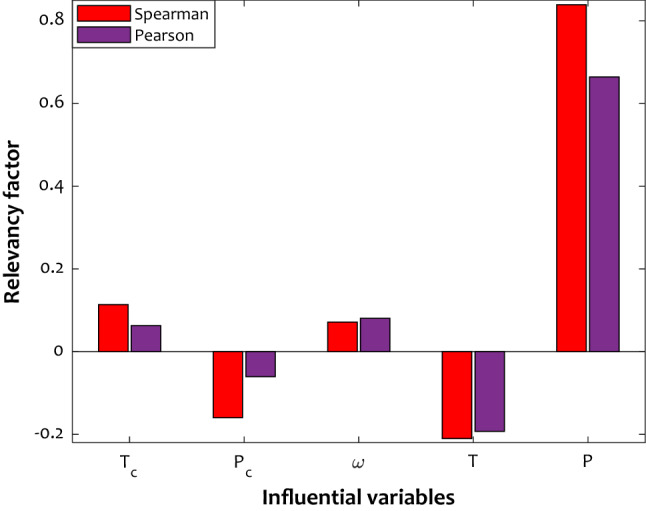
Dependence of H_2_S solubility in ILs on its influential parameters.

As stated earlier, the best model was determined LS-SVM with the input parameters, including pressure, temperature, acentric factor, and critical pressure and temperature. In order to study the influence of input parameters on the dissolved mole fraction of H_2_S in ionic liquids, the relevancy factor was utilized^89^. This relevancy factor (${\text{r}}_{{\text{i}}}$) is defined for all independent variables (i) as follows^90^:

18 $$r_{i} = \frac{{\mathop \sum \nolimits_{k = 1}^{n} \left( {M_{i,k} - \overline{M}_{i} } \right)(N_{k} - \overline{N})}}{{\sqrt {\mathop \sum \nolimits_{k = 1}^{n} \left( {M_{i,k} - \overline{M}_{i} } \right)^{2} \mathop \sum \nolimits_{k = 1}^{n} \left( {N_{k} - \overline{N}} \right)^{2} } }} \left( {i = 1, \ldots ,5} \right)$$

where $M_{i,k}$,$\overline{M}$,$n$, $N_{k}$, and $\overline{N}$ represent input parameters, an average of inputs, number of the data points, output parameter, and average of output, respectively. The value of $r_{i}$ is located within  − 1 to 1, and the large values correspond to the strong correlation. Also, the increasing or decreasing of output parameter with variations in $M_{i}$ attribute to a positive or negative sign, respectively. Two main techniques, namely Spearman and Pearson^91^, relevancy factors were calculated to ascertain the reliability of the interrelation of the considered independent variables with the H_2_S solubility as the model's output. According to the results of both methods (Fig. 8), pressure and temperature have the most significant roles in this process, while the acentric factor has the lowest effect. Moreover, it was found that the H_2_S solubility adversely relates to the temperature and critical pressure of the ionic liquids. Generally, by increasing the pressure, critical temperature, and the acentric factor of ionic liquids, more H_2_S is expected to be captured.

### Trend analysis of the LS-SVM

Besides being precise, the developed LS-SVM approach should be able to detect the physical trend of the simulated phenomenon. For doing so, the LS-SVM predictions for H_2_S solubility in ionic liquids in various temperatures and pressures were compared to the experimentally measured data.

#### The effect of temperature and pressure

**Figure 9:**
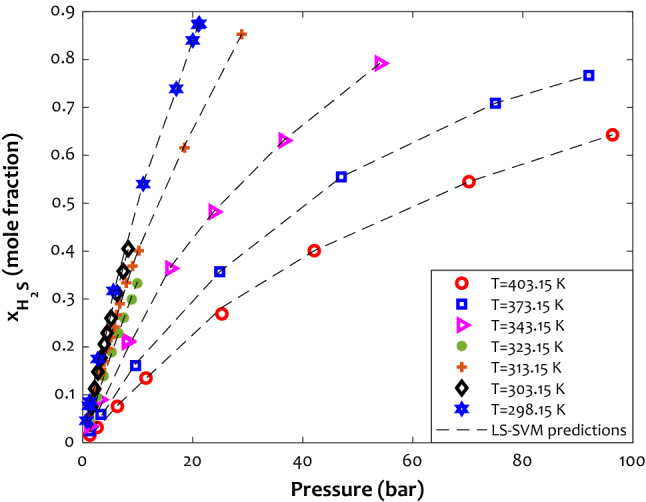
The effect of pressure on the H_2_S solubility in [BMIM][PF_6_] ionic liquid in a wide range of temperatures.

Figure 9 illustrates the solubility of hydrogen sulfide in 1-Butyl-3-methylimidazolium hexafluorophosphate ([BMIM][PF_6_]) in terms of operating pressure at various temperatures. The H_2_S solubility in [BMIM] [PF_6_] was investigated between T = 298.15 K and T = 403.15 K and pressure up to 100 bar. As expected, more H_2_S molecules dissolved in the [BMIM][PF_6_] by increasing the operating pressure. However, the H_2_S solubility in the ionic liquid dramatically decreases as temperature increases. The enhancing effect of pressure is related to the fact that the pressure pushes the H_2_S molecules into the liquid phase^92^. Furthermore, this enhancement is more significant in lower pressure. Increasing the kinetic and internal energy of the hydrogen sulfide molecules by increasing the temperature may be responsible for this observation^92^. Furthermore, dissolving H_2_S in liquid is an exothermic process. When this gas dissolves in ILs, its molecules interact with ionic liquid molecules and release heat within attractive interaction. Conversely, increasing temperature by adding heat to the solution provides thermal energy that overcomes the attractive forces between the gas and the liquid molecules, thereby decreasing the solubility of the gas.

The outstanding performance of LS-SVM for predicting the profile and all distinct data points can be concluded from this figure. Indeed, the proposed LS-SVM model successfully understands the influence of operating pressure and temperature on the hydrogen sulfide solubility in the ionic liquid.

#### The effect of cation and anion type

**Figure 10:**
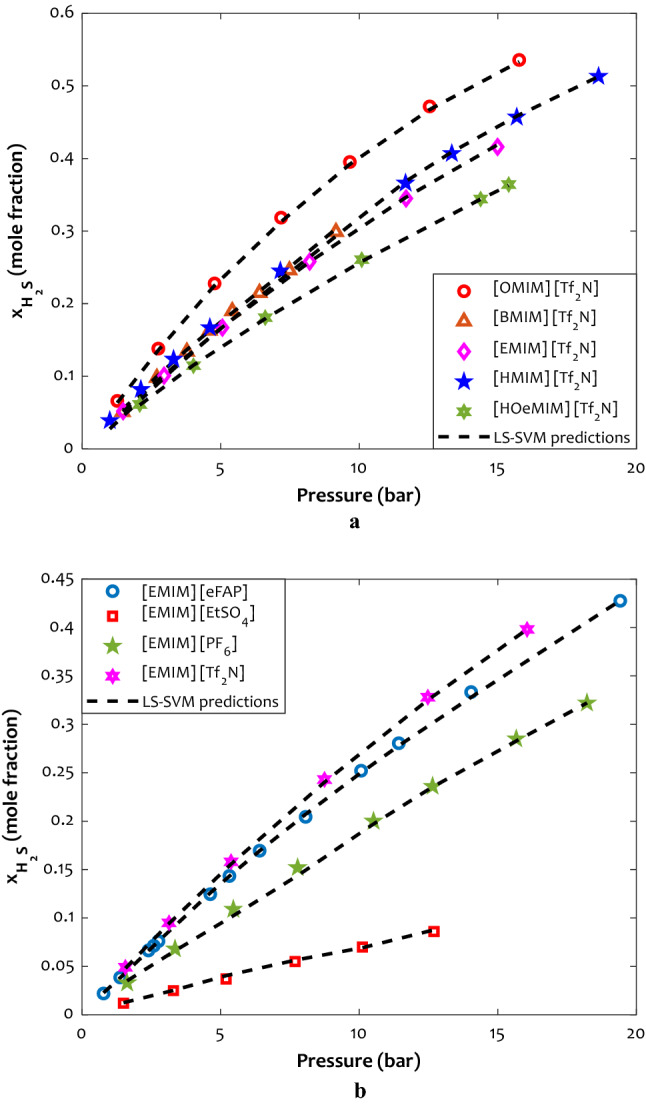
(**a**) The effect of cation type on the H_2_S solubility in [Tf_2_N]-contained ionic liquids (T = 343.15 K) (**b**) The influence of anion type on the hydrogen sulfide capture ability of the [EMIM]-contained ionic liquids (T = 353.15 K).

Assessing the effects of both anions and cations leads to a deep perception of the behavior of H_2_S solubility in ILs. It is generally found that anions have more influence on the solubility of H_2_S gas than cations^93^ As illustrated in Figs. 10a and b, higher H_2_S solubility was obtained for ILs with the same [TF_2_N]^−^ anion but higher alkyl chain length ([C_8_MIM]^+^ > [C_6_MIM]^+^  > [C_4_MIM]^+^  > [C_2_MIM]^+^  > [C_2_OHMIM]^+^). This originates because the longer alkyl chain provides more free volume available in ILs. Aki et al.^94^ ascribed this behavior to entropic rather than enthalpic reasons, where the molar density of the ILs decreases as the length of the cation alkyl chain gets larger^95^. As the molar density of the IL decreases, the free volume of the IL enhances the absorption of H_2_S to occur through a space-filling mechanism^96^. Consequently, larger free volumes increase H_2_S solubility by stronger Van der Waals interactions and more H_2_S molecules absorbed in the solvent^23^. The above-mentioned trend is an outcome of the variations in molecular interactions of H_2_S with ionic liquids, which arise from the differences in the chemical constituents, shapes, and sizes of ILs. In addition, H_2_S solubility for ILs with similar [EMIM]^+^ but different types of anions was investigated. It was found that higher H_2_S solubility obtains in anions containing more fluorine content ([TF_2_N]^−^  > [eFAP]^−^  > [PF_6_]^−^  > [EtSO_4_]^−^), which is in accordance with the other reports in the literature^31^. Moreover, CO_2_ and H_2_S could be strongly attracted in ILs containing [Tf_2_N]^−^ in compassion with [PF_6_]^−^^97^. As can be seen, the LS-SVM model as a non-linear approach possesses the exceptional capability for estimating the H_2_S solubility behavior in terms of anion and cation types to obtain reliable quantitative results.

#### The effect of ionic liquid type (cation + anion)

**Figure 11:**
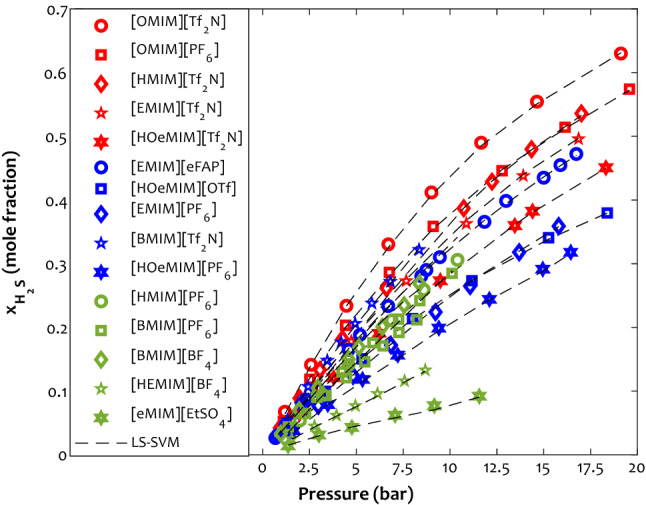
Comparing the H_2_S capture tendency of various ionic liquids at T = 333.15 K.

Previous analyses show that the [OMIM]^+^ and [Tf_2_N]^-^ provide the highest H_2_S absorption capacity for the ionic liquid. This section aims to investigate whether their combination poses the highest absorption capacity or not. The effect of absorbent type (combination of cation and anion) on the H_2_S dissolution in the ionic liquid at the temperature of 333.15 K is depicted in Fig. 11. Generally, an astonishing agreement exists between the calculated hydrogen solubility and the experimentally measured values. As expected, the combination of the [OMIM]^+^ and [Tf_2_N]^-^, i.e., [OMIM][Tf_2_N] ionic liquid, shows the highest tendency for absorbing the H_2_S molecules. On the other hand, the [eMIM][EtSO_4_] is the worst medium for capturing the hydrogen sulfide molecules.

### Maximizing H_2_S solubility in ionic liquid

**Figure 12:**
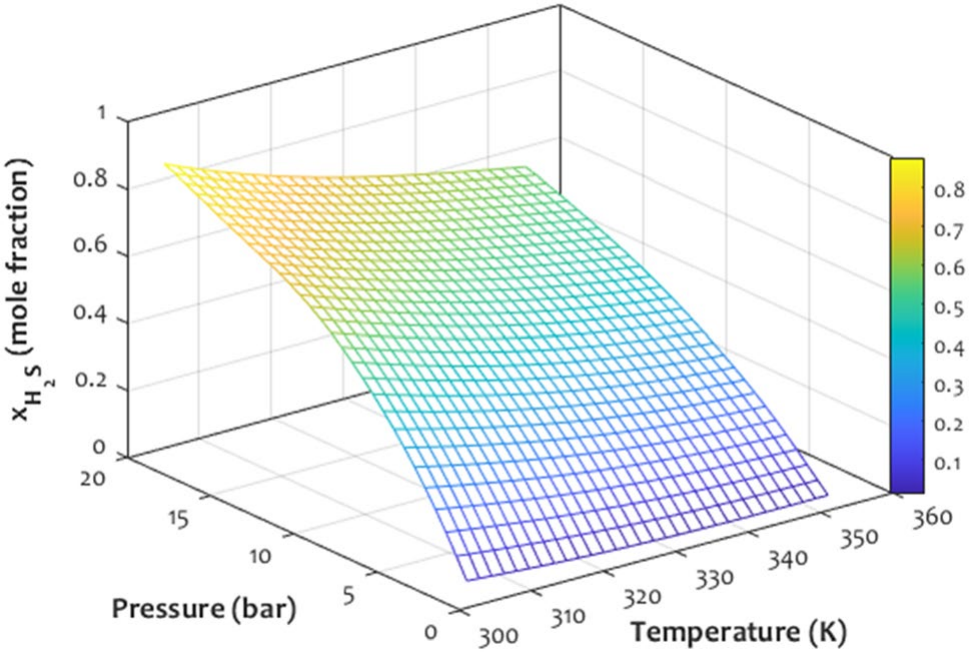
Three-dimensional illustration of temperature and pressure coupled effect on the H_2_S solubility in [OMIM][Tf_2_N].

The previous investigations approved that combining the [OMIM]^+^ and [Tf_2_N]^-^, i.e., [OMIM][Tf_2_N] ionic liquid synthesized the best medium for absorbing the hydrogen sulfide molecules. This section uses the developed LS-SVM approach to graphically determine the operating condition that maximizes hydrogen sulfide absorption capacity of the [OMIM][Tf_2_N] ionic liquid. Figure 12 presents pure simulation results for the effect of simultaneous change of pressure and temperature on the H_2_S solubility in the [OMIM][Tf_2_N] ionic liquid. It can be seen that increasing the pressure and decreasing the temperature gradually increases the H_2_S dissolution in the considered ionic liquid. Therefore, the maximum hydrogen solubility of ~ 0.8 is achievable at the highest pressure and lowest temperature.

### Applicability of LS-SVM and outlier detection

**Figure 13:**
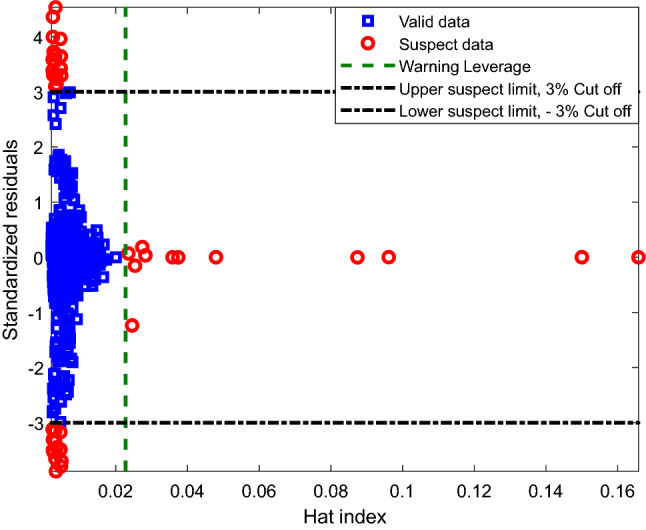
Outlier/valid data detection by the Leverage method.

During the model development, standard residuals were calculated and plotted. Data points with standard residuals in the range of − 3 to + 3 (illustrated on the y axis) and Hat indexes limited to 0. The calculated H* (x-axis) values have been recognized as good data points. William’s plot related to the developed LS-SVM model is shown in Fig. 13. As can be seen in this plot, the significant numbers of the point are located in the good Leverage area (0 ≤ H ≤ 0.022 and − 3 ≤ SR ≤ 3)^98^. Hence, the Leverage approach confirms the validity and reliability of the proposed LS-SVM model for estimating H_2_S solubility in ILs. Furthermore, the number of outliers is too small to affect the modeling generalization negatively.

### Application range of the constructed LS-SVM model

Table 1 shows that the H_2_S solubility data utilized to develop the LS-SVM model are only about imidazole-based ionic liquids containing F atoms. Therefore, this intelligent approach is only valid for the utilized ionic liquids in the reported pressure and temperature ranges.

On the other hand, many different non-F functionalized ionic liquids have also been utilized for H_2_S absorption. It is possible to collect a databank for H_2_S solubility in non-F functionalized ionic liquids (or for both F functionalized and non-F functionalized ionic liquids), develop different machine learning methods, compare their accuracy, and find the most accurate model.

## Conclusion

The absorption process is likely the most widely used method for H_2_S removal. Untapped potentials and favorable characteristics of ionic liquids have been enticing for scientists to investigate their H_2_S removal capacity. However, experimental endeavors are not only costly but time-consuming. On the other hand, since there are many affecting parameters and the interactions between IL and H_2_S molecules are complex, accurate results cannot be achieved by the equations of state. Fortunately, AI methods can bypass theoretical equations and solve complicated problems expeditiously and accurately. The current study investigated H_2_S solubility in fifteen ILs by implementing six robust AI methods, including MLPNN, LS-SVM, ANFIS, RBFNN, CFFNN, and GRNN. The temperature, pressure, acentric factor, critical pressure, and critical temperature of investigated ILs are influential variables of the current study. The validation of the derived models was approved using seven statistical criteria. It was found that the LS-SVM was the best predictive model having R^2^, RMSE, MSE, RRSE, RAE, MAE, and AARD of 0.99798, 0.01079, 0.00012, 6.35%, 4.35%, 0.0060, and 4.03%, respectively. It was found that temperature and the critical pressure of the liquid are adversely related to the H_2_S solubility. However, the pressure, critical temperature, and acentric factor of ionic liquids increase H_2_S dissolution in ionic liquids. The outlier detection method justified that a relatively substantial number of data points are valid and have enough quality to be incorporated into the modeling procedure. Finally, the maximum hydrogen solubility of ~ 0.8 is achievable by [OMIM][Tf_2_N] ionic liquid at the highest pressure and lowest temperature.

## Supplementary Information

Supplementary Information.

## Data Availability

A user-friendly and straightforward Matlab-based code has been prepared to use by other research groups (please see Supplementary Information: supplementary_file\Matlab_code). The collected experimental databank has been added to the revised manuscript (please see Supplementary Information: supplementary_file\Database).
